# The Consequences of Mitochondrial T10432C Mutation in Cika Cattle: A “Potential” Model for Leber’s Hereditary Optic Neuropathy

**DOI:** 10.3390/ijms23116335

**Published:** 2022-06-06

**Authors:** Dinko Novosel, Vladimir Brajković, Mojca Simčič, Minja Zorc, Tanja Svara, Karmen Branovic Cakanic, Andreja Jungić, Betka Logar, Vlatka Cubric-Curik, Peter Dovc, Ino Curik

**Affiliations:** 1 Department of Pathology, Croatian Veterinary Institute, 10000 Zagreb, Croatia; branovic@veinst.hr; 2 Department of Animal Science, Faculty of Agriculture, University of Zagreb, 10000 Zagreb, Croatia; vbrajkovic@agr.hr (V.B.); vcubric@agr.hr (V.C.-C.); 3 Department of Animal Science, Biotechnical Faculty, University of Ljubljana, 1000 Ljubljana, Slovenia; mojca.simcic@bf.uni-lj.si (M.S.); minja.zorc@bf.uni-lj.si (M.Z.); peter.dovc@bf.uni-lj.si (P.D.); 4 Institute of Pathology, Wild Animals, Fish and Bees, Veterinary Faculty, University of Ljubljana, 1000 Ljubljana, Slovenia; tanja.svara@vf.uni-lj.si; 5 Department of Virology, Croatian Veterinary Institute, 10000 Zagreb, Croatia; jungic@veinst.hr; 6 Agricultural Institute of Slovenia, 1000 Ljubljana, Slovenia; betka.logar@kis.si

**Keywords:** animal model, retinal ablation, cattle, detrimental mutations, LHON, mitogenome

## Abstract

While mitogenome mutations leading to pathological manifestations are rare, more than 200 such mutations have been described in humans. In contrast, pathogenic mitogenome mutations are rare in domestic animals and have not been described at all in cattle. In the small local Slovenian cattle breed Cika, we identified (next-generation sequencing) two cows with the T10432C mitogenome mutation in the ND4L gene, which corresponds to the human T10663C mutation known to cause Leber’s hereditary optic neuropathy (LHON). Pedigree analysis revealed that the cows in which the mutation was identified belong to two different maternal lineages with 217 individual cows born between 1997 and 2020. The identified mutation and its maternal inheritance were confirmed by Sanger sequencing across multiple generations, whereas no single analysis revealed evidence of heteroplasmy. A closer clinical examination of one cow with the T10432C mutation revealed exophthalmos, whereas histopathological examination revealed retinal ablations, subretinal oedema, and haemorrhage. The results of these analyses confirm the presence of mitochondrial mutation T10432C with homoplasmic maternal inheritance as well as clinical and histopathological signs similar to LHON in humans. Live animals with the mutation could be used as a suitable animal model that can improve our understanding of the pathogenesis of LHON and other mitochondriopathies.

## 1. Introduction

Mitochondrial DNA (mtDNA), which occurs in hundreds of copies per cell, encodes 13 of the ~85 components of oxidative phosphorylation (OXPHOS), a metabolic pathway critical for aerobic respiration [1]. The present-day vertebrate mitogenome is significantly reduced, as much of it has been relocated to the nucleus during evolution. Therefore, genes in the mitogenome interact closely, with about one to two thousand “ex-mitochondrial” genes located on nuclear DNA. Inheritance of mitochondrial DNA follows a non-Mendelian pattern, as the mitogenome is passed on maternally without recombination in vertebrates, while the mutation rate is much higher compared to nuclear DNA.

Some mitogenome point mutations can alter the polarity and structure of the encoded enzyme and block the binding of a specific enzyme in the OXPHOS process, with disastrous consequences for cellular metabolism. In human medicine/genetics, a number of diseases caused by dysfunctional mitochondria have been identified and well-documented. In about 15% of humans, mitogenome mutations can lead to clinical symptoms such as poor growth, loss of muscle coordination, muscle weakness, visual and hearing problems, learning disabilities, heart disease, and dementia [2]. The most important diseases in humans are mitochondrial myopathy, encephalopathy, lactic acidosis, stroke-like symptoms (MELAS), Leber’s hereditary optic neuropathy (LHON), or Leigh syndrome (also known as Leigh disease, LD) [3,4]. LHON is one of the first, most important, and best-studied mitochondrial diseases, affecting largely young men and characterised by subacute/acute loss of central vision, possibly leading to blindness. Interestingly, there is a number of LHON variants, each associated with different point mutations [5,6,7] in the mitogenome, mainly mutations in the ND1, ND3, ND4, ND4L, ND5, and ND6 genes

In general, mitogenome mutations leading to pathological manifestations such as LHON are rare. In contrast, spontaneous pathogenic mitogenome mutations are extremely rare in domestic animals. Nevertheless, two cases with pathological consequences of maternally inherited mitogenome mutations have been described in dogs. One is mitogenome mutation G14474A, which results in a valine-to-methionine amino acid change at position 98 of the cytochrome b gene and causes various states of progressive neurological degeneration with spongiform encephalomyelopathy—a disorder with symptoms similar to Kearns–Sayre syndrome in humans [8]. The other is sensory ataxic neuropathy in golden retrievers caused by a deletion of one base pair in the mitochondrial tRNATyr gene [9]. However, while there are several good animal models in which nuclear mutations mimic the pathological state of mitochondrial disorders in humans [10,11,12], there was a comparative dearth of good animal models in which mitogenome mutations are the primary cause of disorders, largely due to technical difficulties [13].

Recently, a large-scale study of 799 complete mitogenomes from more than 100 breeds identified two distinct mutations, both associated with point mutations in the mitogenome that are also present in humans (C4171A in the ND1 gene and T10663C in the ND4L gene) and linked to LHON [14]. For example, one Croatian Busha cattle animal had mitogenome mutation C3965A (C4171A mutation in humans), while two Slovenian Cika cattle animals had mutation T10432C (T10663C mutation in humans). However, Novosel et al. (2019) [14] only reported mutations and a clinical report of exophthalmos in one cow, although this was an interesting unique finding and a potentially nice animal model for better insight into the LHON pathology. In humans, the T10663C mutation is located in the ND4L gene and is coinherited by all maternally related males and females. The consequence of the mutation is a change in the amino acid sequence of the ND4L protein at codon 65 from valine to alanine (Val65Ala) [15]. The specific mutation has been observed only as a result of homoplasmy, without evidence of heteroplasmy [16], and causes colour blindness, disc oedema, retinal ganglion cell death, and optic nerve atrophy [15]. Since the consequences of the LHON mutation in the ND4L gene itself are not well-documented in humans, e.g., there are no available histopathological reports, a more comprehensive analysis of the T10432C mutation is needed. In general, this would be a unique description of the mitogenome point mutation showing a similar pathology in non-experimental animals and humans.

Thus, the main objective of this study was to extend the findings of Novosel et al. (2019) [14] to the inheritance and pathological consequences of the T10432C mitogenome mutation (T10663C mutation in humans) in the Cika cattle. Here, we focus on the following analyses: (i) confirmation of the maternal inheritance of the T10432C mutation over multiple generations, (ii) analysis of whether the mutation is homoplasmic or heteroplasmic, (iii) detailed histopathological analysis of the eye bulb and brain, (iv) identification of the potential number of “mutant” animals in the breed, and (v) comparison of animals within the mutation lineage(s) to other animals with respect to various traits important for breeding the Cika cattle.

## 2. Results

### 2.1. Identification of “Mutant” Individuals and Homoplasmic Maternal Inheritance

**Figure 1:**
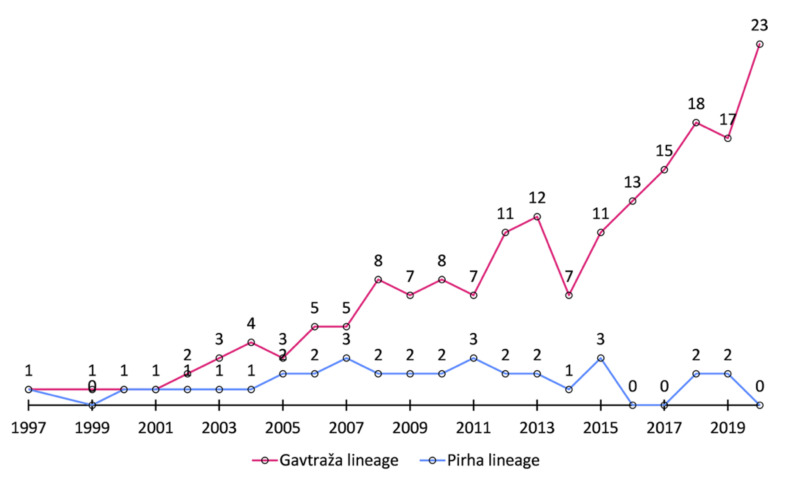
The number of animals born from 1997 to 2000 in the “mutant” lineages.

Pedigree analysis shows that the two “mutant” cows, Gavtraža and Pirha, were found in two maternal lines that are not connected by the pedigree. In total, we found 217 animals born between the years 1997 and 2020 in these two lineages. Interestingly, the number of individuals in the Gavtraža line has been increasing in absolute numbers, reaching 1.6% (23/1432) of the Cika animals born in 2020 (see Figure 1), indicating that mutant animals do not show a significant reduction in their production performance. At this point, we should emphasize that the diagnosis of exophthalmos in Gavtraža was made after the identification of the “mutant” mitogenome sequences and not vice versa. Moreover, Gavtraža was a respectable productive cow that lived a long life and left many offspring.

**Figure 2:**
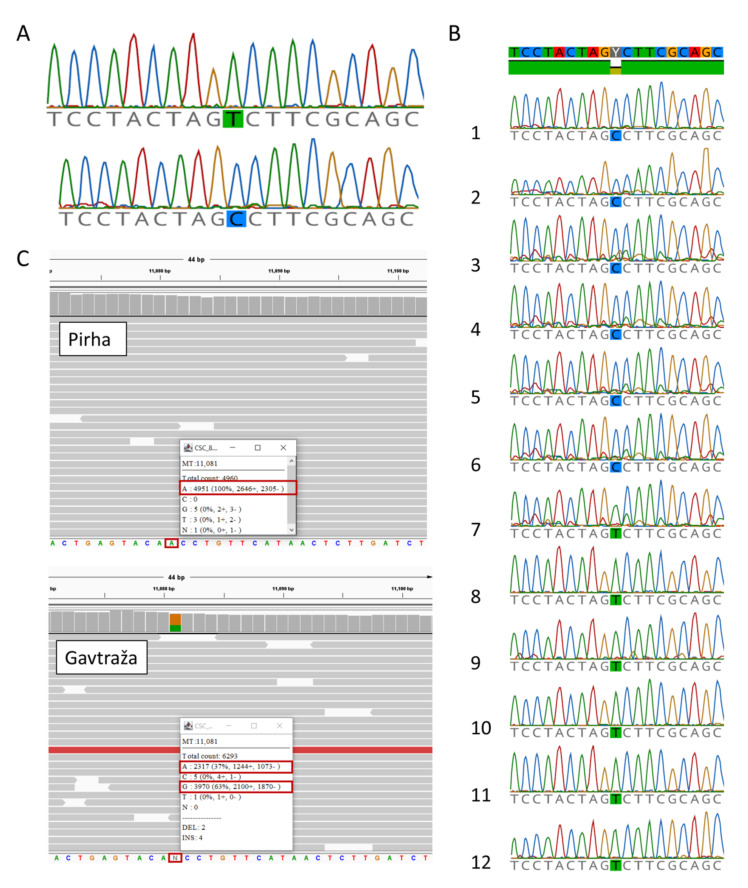
Sequencing of mtDNA revealed two mutations in Cika cattle. (**A**) Homoplasmic mutation T/C at mtDNA position 10,432 confirmed by Sanger sequencing. (**B**) The mutation (C allele) was found in two maternal lineages (Gavtraža and Pirha). The wild type (T allele) was present in all individuals not maternally related to Gavtraža and Pirha. Sequences of mutant and wild-type mtDNA around position 10432: (B1) Gavtraža’s sequence from the muscle tissue; (B2) Gavtraža’s sequence from the brain; (B3) Breza’s sequence; (B4) Košuta’s sequence; (B5) Cow 9502′s sequence; (B6) Cow 9505′s sequence; (B7) Holstein–Friesian control sample’s sequences; (B8–B12) Cika individuals not maternally related to Gavtraža and Pirha. All the sequences shown from B3 to B12 were obtained from blood cells. (**C**) NGS sequencing of the whole mitochondrial genome revealed an additional heteroplasmic mutation in Gavtraža (A/G) at position 11,081 bp, but not in Pirha.

When we compared the complete mitogenome sequences of Gavtraža and Pirha, they were almost identical with only a negligible difference. More precisely, we detected a single heteroplasmy in the mitogenome sequence of Pirha, where nucleotide A occurs 2317 times at position 11081, in contrast to nucleotide G which occurs 3970 times. This heteroplasmy, the appearance of A and G, was observed at the third codon position and was synonymous—causing no amino acid change (Figure 2). Thus, the probability that these two mutations have the same origin, i.e., that they represent the same “mutation line” (T10432C), is extremely high.

**Figure 3:**
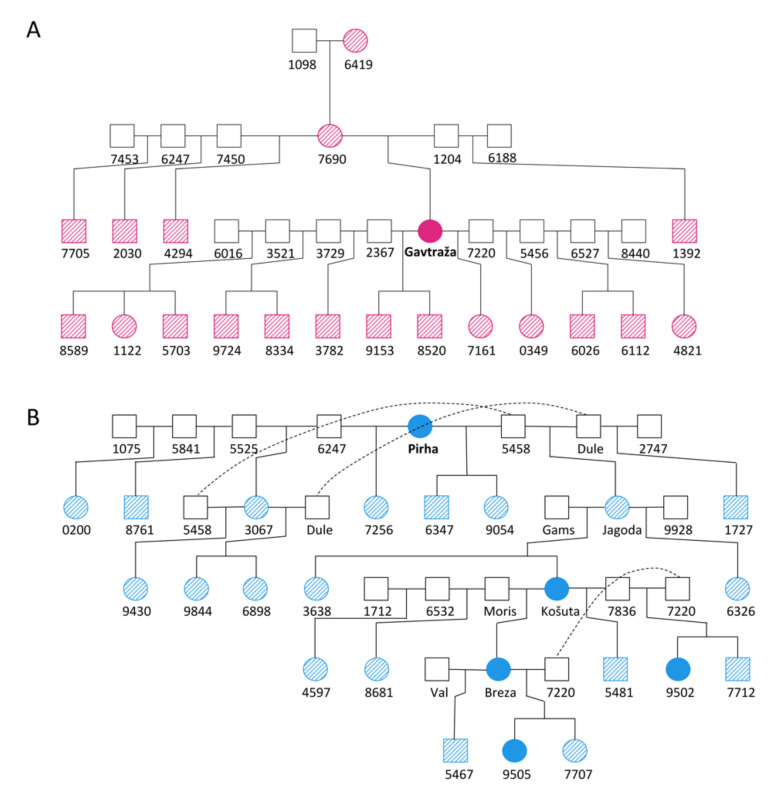
Reduced pedigrees of two Cika cows with identified T10432C mtDNA mutation. (**A**) Pedigree of cow Gavtraža and (**B**) pedigree of cow Pirha. Solid colour represents individuals with a genotyped mutation. Individuals carrying the mutation (based on pedigree data) are shown with diagonal lines.

Maternal inheritance was confirmed by Sanger sequencing, with individuals carrying the T10432C mutation for five generations (Figure 3). The evidence of maternal inheritance of the mutation is even more reliable considering that the presence of the T10432C mutation in Gavtraža and Pirha most likely has the same origin. At the same time, the results of next-generation sequencing and Sanger sequencing (see Figure 2) show no evidence of heteroplasmy at mitogenome position 10432, confirming that the identified mutation is homoplasmic.

### 2.2. Clinical and Histopathological Examinations

**Figure 4:**
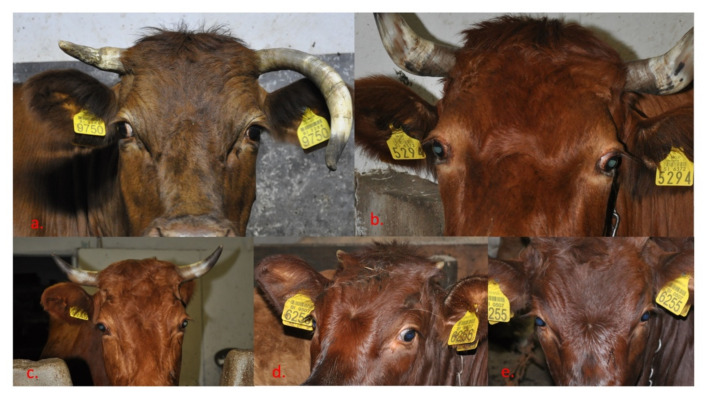
Eyes of the cows—carriers of the mutation: (**a**) Gavtraža with exophthalmos of the right eye, (**b**) Košuta with weak exophthalmos of the right eye and moderate exophthalmos of the left eye; (**c**) Breza with weak exophthalmos of the right eye, (**d**) a heifer with no signs of exophthalmos, (**e**) a heifer with no signs of exophthalmos (photographs: Simčič M.).

The animal was examined by clinical inspection and looked as if it was shocked or impressed, the bullae were pushed forward, and the white part was more prominent. Ocular protrusion was mostly visible unilaterally, while irritation was not observed. Clinical findings were consistent with exophthalmos. Exophthalmos was found in the examined cow Gavtraža (Figure 4a), but also in another old cow Košuta (Figure 4b), while three other cows (Breza (Figure 4c), unnamed (Figure 4d), and unnamed (Figure 4e)) were without clinical manifestations.

**Figure 5:**
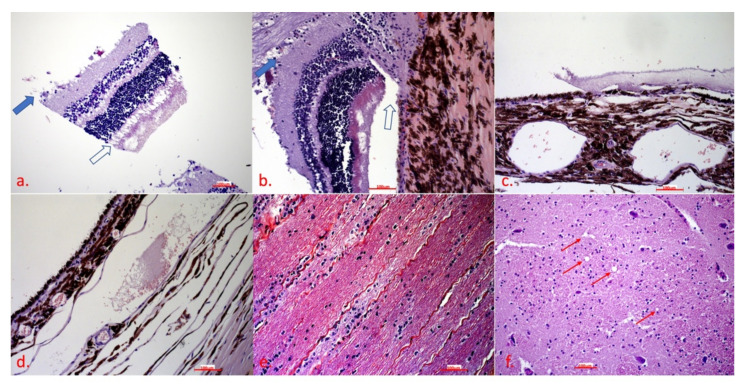
Histopathological examination reveals significant lesions in the eye bulb: (**a**) retina—the bold arrow shows apoptosis of the ganglion cell layer, the blank arrow shows apoptosis of the rod and cone layer; (**b**) optic disc—the bold arrow shows apoptosis of the ganglion cell layer and the optic nerve, the blank arrow shows ablation of the retinae; (**c**) subchoroidal layer—oedema causing retinal ablation; (**d**) choroidal layer—oedema and haemorrhage; (**e**) optic nerve—apoptosis within the optic nerve and microgliosis as a reparative process; (**f**) brain, spongiform-like lesion—the red arrows show vacuoles. In all the figures from (**a**) to (**f**), the scale bar corresponds to 100 μm (20× magnification). H&E.

**Figure 6:**
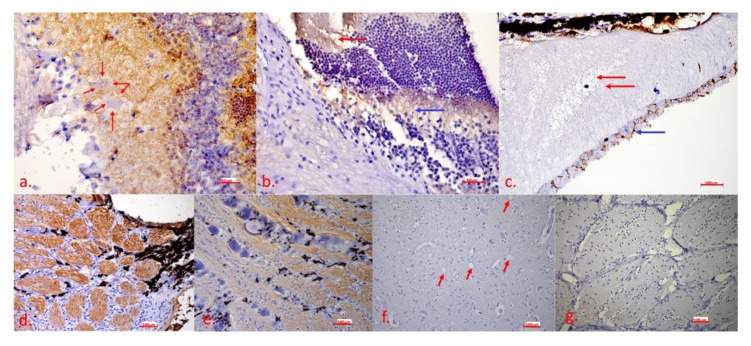
IHC detection of caspase 3 shows the presence of apoptosis in the eye bulb and the brain: (**a**) retina—strong presence of the brown chromogen in the inner plexiform layer, presence of the brown chromogen in the ganglion cells (red arrows); (**b**) optic disc—presence of the brown chromogen in the cone and rod layer (red arrow) and the outer plexiform layer (blue arrow); (**c**) subchoroidal layer/retina—oedema (red arrow) between the pigment epithelium and the cone and rod layer, presence of the brown chromogen in the cone and rod layer (blue arrow); (**d**) optic disc—strong presence of the brown chromogen in nerve fibres; (**e**) optic disc—calcifications between nerve fibres; (**f**) brain—no apoptosis signalling, vacuoles are marked with the red arrow; (**g**) optic disc—negative control. In Figure 6a,f, the scale bar corresponds to 50 μm (40× magnification), in Figure 6b,c,d,e,g, the scale bar corresponds to 100 μm (20× magnification). IHC to detect caspase 3, DAB chromogen, counterstain with Mayer’s haematoxylin.

Severe apoptosis and oedema were observed in several layers. Apoptosis was most pronounced in the ganglion cell layer (Figure 5a and Figure 6a, the inner plexiform layer (Figure 6a), the outer plexiform layer (Figure 6b), the rod and cone layer (Figure 5a,c and Figure 6b), and the optic nerve (Figure 5b,e,d) with calcifications (Figure 6e). Severe oedema with haemorrhage leading to retinal ablation was located in the subchoroidal layer (Figure 5c,d and Figure 6c). In the brain, there was no apoptosis signalling (Figure 5f and Figure 6f), and even indeterminate spongiform-like lesions (Figure 6f) were observed.

Eye bulbs were removed immediately after slaughter and placed in a fixative. Immediately after fixation, the retina was observed to detach and partially float in the fixative and partially float in the vitreous. Histopathological examination revealed a marked discontinuity of layers, with the retina actually held by the orbit only in the region of the optic disc, while in other parts it floated in the vitreous and was only partially attached zonally. In the area between the retina and the deeper parts in the areas where it begins to detach, oedematous fluid oozing, weak eosinophilic staining, but also accumulations of erythrocytes that certainly should not have been there were observed. The morphologic destruction of the nervous tissue was particularly marked. The ganglion cell layer was largely absent as the cells were completely disintegrated, with only cellular debris visible. Strong caspase 3 signalling was also present in this layer, indicating strong apoptosis. Even in the still-vital ganglion cells, there was apoptosis signalling in the cytoplasm. Apoptosis was present in the inner and outer plexiform layers without visible morphological changes.

Most conspicuous were subretinal oedema and haemorrhages separating the subchoroidal layer and the retina. There were severe death, microgliosis, and calcium deposition in the optic disc and at the beginning of the optic nerve, suggesting that this process was prolonged and may have begun to manifest there. It is very likely that this animal had severe vision problems. It is likely that this health problem is hidden because the history data lack any information about health problems, even vision problems. Finally, the owners would not have kept the animal until it had health problems but would have gotten rid of it much earlier.

### 2.3. Suitability of “Mutant” Cattle for Cika Cattle Breeding Goals

**Figure 7:**
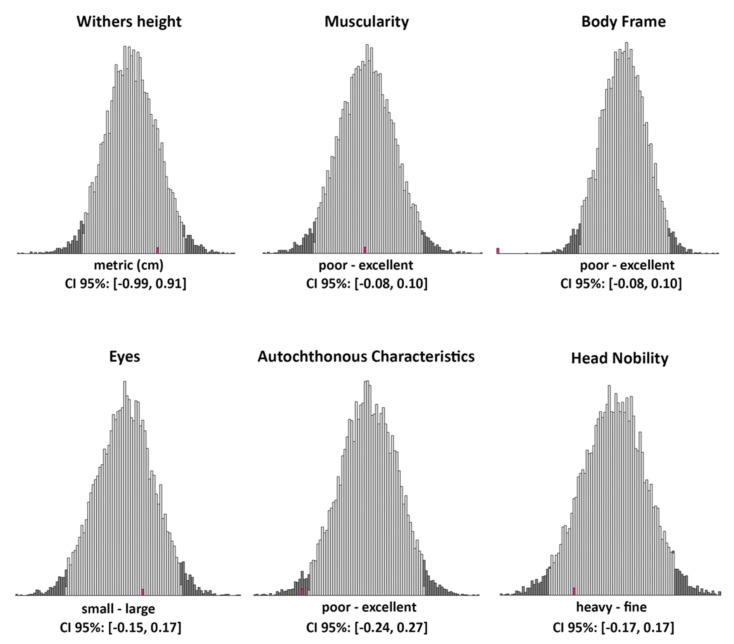
Distributions of the mean breeding values for the sample of 44 animals without the “mutation” (10,000 samples) with credibility intervals (grey bars) for six type traits important in the breeding of Cika cattle. The mean of the breeding values for 44 animals with the “mutation” is shown in red.

The increase in the number of animals with the T10432C mutation apparently indicates that the negative consequences of the mutation, which were not observed or present, did not influence the breeder’s decision to continue breeding these animals. However, using a sample of six type traits used as selection criteria in Cika cattle, we wanted to know if animals with the “mutation” fit well into the breed’s breeding goals (Figure 7). To answer this question, we compared the mean breeding values (BV) of the sample of 44 animals from maternal lineages with the mutation with the distribution of the mean breeding value of the sample of 44 animals drawn 10,000 times from a dataset of 3578 animals originating from maternal lineages without the mutation. As shown in Figure 7, the mean breeding values of our “mutation” sample were within the credibility interval (95%) of the generated distribution for withers height (mean BV = 0.41), muscularity (mean BV = 0.01), eyes (mean BV = 0.06), and head nobility (mean BV = −0.10). This result shows that, from the breeding point of view, animals with the mutation are acceptable for further breeding. However, for two traits, autochthonous characteristics (mean BV = −0.27) and body frame (mean BV = −0.25), the observed mean BVs were not within the 95% credibility interval.

## 3. Discussion

In this study, we extended the findings of Novosel et al. (2019) [14], in which exophthalmos was observed in a Cika cow with mitogenome mutation T10432C. This mutation corresponds to the T10663C mutation in humans, which is known to cause LOHN. We confirmed maternal inheritance of the T10432C mutation over multiple generations because all the other sequenced offspring of the “mutant” cows had the T10432C mutation. We did not detect evidence of heteroplasmy in any of our molecular analyses, whereas homoplasmic inheritance of the T10432C mutation was observed in animals descended through multiple generations.

The initial anamnestic data were encouraging as this lineage of Cika cattle is known for its “bulging eyes”, possibly indicating the presence of exophthalmos. In the first animal carrying the deleterious mutation, exophthalmos was already detected during a clinical examination. Using pedigree analysis, we were able to identify a large number of other maternally related animals, where we also detected the presence of exophthalmos in four clinically examined “mutant” cows. Of course, the first identified animal called Gavtraža was quite old and therefore continued to be observed until the breeder decided to send it to the slaughterhouse. This was a good opportunity for more detailed clinical and histopathological examinations.

The comparisons made showed that the breeding values for withers height (cm), muscularity (poor to excellent), eyes (small to large), and head nobility (heavy to fine) of the “mutant” animals were within the same expected range (95% credibility interval) as for animals without an identified mutation. In contrast, the observed breeding values for body frame (poor to excellent) and autochthonous characteristics (poor to excellent) in the “mutant” 44 individuals were much less desirable than expected in the samples with “normal” individuals (on the left side of the 95% credibility interval). More detailed analyses revealed that large body size was the main reason for the observed result, as large body-framed animals are less preferred in Cika cattle breeding [17]. Therefore, large animals would be evaluated with poor body frame and poor autochthonous characteristics. However, from the functional and biological points of view, large animals are considered healthy, so we cannot draw any negative associations in relation to the T10432C mutation and the six analysed traits.

Histopathological examination revealed very specific lesions in the bulb of the eye, consisting of retinal ablation by oedema secondary to apoptosis of several layers of the eye and, in particular, of the ganglion cell layer and the optic nerve. Immunohistochemistry confirmed apoptosis. The character of the lesions was compatible with the previously described lesions caused by LHON [18].

One of the objectives of this study was to compare animals within the mutation line(s) with other animals with respect to six type traits that are representative and important for the breeding of Cika cattle. Our comparison was based on the BVs of 44 “mutant” compared to 3578 “normal” first-parity cows, all born between 1997 and 2020. BVs were predicted in the first national breeding value evaluation in the year 2022 for the Cika breeding program. In addition, Simčič et al. (2015) [19] reported the presence of old admixture in a period of Cika cattle breeding. In the current breeding program of the Cika cattle, large cattle would be at a disadvantage, especially for some type traits (body frame and autochthonous characteristics) (see Simčič et al. (2021) [17]).

Regarding the effects of the mitogenome mutation, our comparison contains several assumptions (aspects) that could influence our conclusions and should be interpreted with caution, taking into account all possible pitfalls. For example, the evaluation of type traits was performed only in first-parity cows and young candidate bulls. Based on 9,525 culls (5931 bulls and 3594 cows) recorded between 2005 and 2020, we found that the life expectancy of bulls (1.6 years, with the oldest male animal reaching an age of 7.5 years) was considerably lower compared with cows (4.1 years, with the oldest cow reaching an age of 22.6 years). LHON is age-dependent, and the effects of the mutation are thought to be more pronounced in older animals. Therefore, it is possible that the identified T10432C mutation affects male animals more severely. However, this was not observed because the life expectancy of the male animals was too short to develop severe symptoms. Breeding values reflect only the additive polygenic component of the analysed traits, which was estimated here using pedigree analysis. Possible direct mitogenome effects or interactions between the mitogenome and additive nuclear effects were therefore not considered but could influence the results. Possibly, more complex analyses targeting mitogenome effects [20] or SNP arrays targeting nuclear genes interacting with the mitogenome [21] would lead to more accurate conclusions. On the other hand, we accounted for possible confounding by age structure in our analysis because 44 “mutant” cows were born equally between 1997 and 2020, which adequately reflects the structure of the sample population (“normal” animals). However, we do not believe that there was a strong functional negative effect of the identified mutation as the number of cattle with the mutation increased over time and the breeders who kept “mutant” cattle did not recognise it or even report additional health problems. It is important to note that the most affected cow, Gavtraža, was almost 16 years old at the time of weaning, which is an extremely advanced age for a cow. In intensive production systems, high-performance cows can live up to 4–7 years during lactation. This certainly does not mean that these animals have poor production performance, but the opposite.

Since the first report by Leber in 1871 [22], there has been a great interest in LHON. In 1980, maternal inheritance was recognised [23], and in 1988 a point mutation in mtDNA was found responsible for this disease [24]. The optic nerve appears to be highly dependent on mitochondrial dysfunction, and a broad category of optic nerve diseases has been recognised to be due to mitochondrial dysfunction [5,25]. Mitochondria play a central role in the optic nerve pathology and retinal ganglion cell death [26,27]. LHON is by far the most common mitochondrial disease in humans [28], and its two components are particularly susceptible to OXPHOS impairment of the pigment/photoreceptor layers [29] and the ganglion cell/nerve fibre layers [30]. LHON remains asymptomatic until patients have blurred or clouded vision on one side, which later becomes bilateral in most cases. It is very difficult to alert an animal’s owner that the animal has a problem with blurred vision in the stall. A number of basic tests should be performed to confirm the diagnosis, including some very specific and advanced methods that are not practical or available in veterinary medicine. Therefore, it is likely that the owner will not notice or report the animal’s possible vision problem. There are not many reports of histopathological lesions in the eye, only a few, and not in the LHON associated with the T10663C mutation.

In summary, we showed that the T10432C mitogenome mutation in the ND4L gene corresponding to the human T10663C mutation known to cause LHON is homoplasmic and maternally inherited across several generations. Our analysis suggests that the identified mutation has a single origin and represents a single maternal lineage from 217 animals born between 1997 and 2020. At the same time, we did not detect any serious functional problems in the cows of the mutant lineages, except for their large body size, while their owners did not report any other health problems. On the other hand, closer clinical examination of one cow (Gavtraža) with the T10432C mutation confirmed exophthalmos as a clinically visible symptom of the mutation, whereas histopathological examination revealed retinal ablation, subretinal oedema, and haemorrhage.

ND4L is a gene of the mitochondrial genome that encodes the NADH-ubiquinone oxidoreductase chain 4L (ND4L) protein. Mutations in ND4L can reduce mitochondrial respiratory chain activity/efficiency. Variants of human ND4L are associated with Leber’s hereditary optic neuropathy (LHON). Two adjacent mutations in human mtDNA that cause a nonconservative amino acid change in the ND4L subunit at codon 47 (Ile47Thr) and codon 65 (Val65Ala) were described in a large family in Kuwait [15]. The sex-specific expression of LHON in this particular pedigree was consistent with the maternal transmission model and male affection. In addition to sex- and age-related determinants of LHON expression, the variability of the disease is determined by the interplay of mtDNA defects, haplogroups, and environmental influences. We did not confirm the direct dysfunction of the OXYPHOS system that caused the point mutation as a consequence as the key elements of the disease indicative of LHON, and no arguments against it.

However, the presented case of a spontaneous mitogenome mutation resembling LHON and a large number of identified live animals seems to be suitable to improve our understanding of the pathogenesis of LHON and other mitochondriopathies. Knowing that developing a good mitogenome mutation model is difficult is of particular value and potential utility. The presence of the identified mtDNA mutation in a larger number of animals of the Cika breed and the availability of biopsy material from these animals at the time of culling make this potential natural model superior to the attempt to create a similar model by genetic engineering. To further confirm the causality of the mitochondrial T10432C mutation in cattle, an analysis of the transmission of the mutation to the offspring of the affected cow will be performed. In addition, a more detailed phenotypic and histological description of the mutation-bearing animals is required.

## 4. Materials and Methods

### 4.1. Identification of the T10432C Mutation, Histopathology Sampling, and Pedigree Analysis

This study is based on the dataset of a large-scale analysis of 797 complete mitogenomes of modern cattle [31] and on the extension of our previous work [14] that identified the T10432C mutation (“mutant“ mitogenome) in two Cika cows called Gavtraža and Pirha. Sequencing of all eight Cika cattle animals presented here was performed using three long-range PCRs, limiting the likelihood of NUMT occurrence. More detailed information on the next-generation sequencing procedure for two “mutant” Cika cattle animals (GenBank acc. Nos. MZ901663 and MZ MZ901663) and six animals representing “normal” complete mitogenomes of the Cika breed (GenBank acc. Nos. MZ901657–MZ901662) was described in [31], whereas the identification of the T10432C mutation was described by Novosel et al. (2019) [14]. In addition, we compared the similarity of two complete “mutant” mitogenomes and analysed the presence of possible heteroplasmy using IGV software (Broad Institute, UCLA).

To identify “mutant” maternal lineages and their members, we analysed Cika cattle pedigree data using MaGelLAn 1.0 software [32]. This analysis allowed us to sample living members of the “mutant” lineages and confirm their maternal inheritance. Fortunately, we were able to sample four offspring of “mutant” cow Pirha, including her daughter and granddaughter (Figure 3). All of these animals were examined clinically, while tissue samples were collected in ethanol to perform PCR and partial sequencing of the mitogenome.

### 4.2. Molecular Analyses

#### 4.2.1. DNA Extraction

Total genomic DNA was extracted from peripheral blood using E.Z.N.A.^®^ Tissue DNA Kit (Omega Bio-Tek, Norcross, GA, USA) according to the manufacturer’s recommendations.

#### 4.2.2. Primer Selection and Polymerase Chain Reaction

The forward (5′-gcattcacagtatctcttgtagga-3′) and reverse (5′-tggatagggagtcggagaaa-3′) primers for amplification of the targeted region of the mitochondrial ND4L gene were selected using Primer 3 software using the reference bovine mtDNA sequence (*Bos taurus* mitochondrion, GenBank acc. No. NC_006853). The 447 bp long fragment of the ND4L gene containing the T10432C mutation site was amplified by polymerase chain reaction (95 °C, 3 min, 30 cycles (95 °C, 30 s; 55 °C, 30 s; 72 °C, 45 s); 72 °C, 5 min; storage at 4 °C). The PCR products were separated by agarose gel electrophoresis.

#### 4.2.3. Sanger DNA Sequencing

The sequencing reaction was performed using 3 µL of a PCR product and BigDye^®^ Direct Cycle Sequencing Kit (Thermo Fischer Scientific, Carlsbad, CA, USA) according to the manufacturer’s recommendations. Sequencing was performed using an ABI 3500 gene analyser. DNA sequences were analysed using Geneious Prime software.

### 4.3. Clinical and Histopathological Examinations

The opportunity to perform clinical and histopathological examinations arose when the owner of the “mutant” cow called Gavtraža decided to send her to the slaughterhouse. Gavtraža was born on 4 April 2004, and was slaughtered on 20 November 2019, when it was 15 years and 7 months old. The cow had a total of 13 lactations, four female calves and nine male calves. The animal was clinically examined. At slaughter, samples of the brain and eye tulip were collected in 10% buffered formalin and stored at +4 °C for 48 h. The tissue was dehydrated in ethanol and xylene of varying quality and embedded in paraffin blocks; 4 μm tissue sections were stained with a standard H&E stain and examined under the microscope.

Immunohistochemical labelling of the caspase 3 antigen to determine its expression level as a specific cell marker of apoptosis was used. The eye bulb and brain sections were deparaffinized with xylene and rehydrated with graded alcohols. Endogenous peroxidase activity was blocked by incubation with 3% hydrogen peroxide in 0.1 M Tris-buffered saline (TBS, pH = 7.6) for 30 min. Specific pre-treatment was performed with citrate buffer (pH = 6) for 20 min at 96 °C. The nonspecific reaction was blocked with 3% bovine serum albumin for 60 min at room temperature. The tissue sections were incubated with a rabbit monoclonal antibody against caspase 3 (cat. No. 9664, Cell Signalling, Danvers, MA, USA, 1:2000) at a dilution of 1/300 and stored at 4 °C overnight. A rabbit polymer HRP-conjugated detection system (Dako, Glostrup, Denmark) with a final 3 min incubation in diaminobenzidine (DAB)–hydrogen peroxide solution (Dako, Glostrup, Denmark) was used to visualize positive reactions. The slides were counterstained with Mayer’s haematoxylin, dehydrated, covered with a microscope slide, and examined microscopically. Lymph nodes from pigs suffering from multisystemic postweaning wasting syndrome served as positive controls. A brain and an eye bulb from a calf suffering from septicaemia and parallel serial sections of paraffin blocks from an examined Cika cow served as negative controls using the same procedure, except that a primary antibody was added.

### 4.4. Suitability of “Mutant” Cattle for Cika Breeding Policy

Cika is an indigenous Slovenian breed whose main breeding goal is to preserve the original phenotype and avoid inbreeding. Therefore, recording of type traits for first-parity cows and estimation of related genetic parameters such as heritability, genetic correlation, and breeding value is routinely performed (Simčič et al., 2021) [17]. To analyse how well animals with the T10432C mutation meet the breeding goal for Cika cattle, we compared the breeding value of cows with the T10432C mutation to cows without this mutation. For the comparison, we selected four individual traits (withers height, cm; eyes, small–large; body frame, poor–excellent; head nobility, heavy–fine) and two composite traits (autochthonous traits, poor–excellent; muscularity, poor–excellent). All six selected traits are inherited as complex traits with a substantial additive polygenic component. The estimated genetic parameters can be found in the article by Simčič et al. (2021) [17]. Our analyses were based on the comparison of available breeding values, 44 animals with the T10432C mutation in the lineage (“mutant” animals) versus 3,578 animals without the mutation in the lineage (“normal” animals). The breeding values are the equivalent of polygenic risk values in human genetics [33,34] and reflect the value of each trait in relation to the breeding goals for Cika cattle. At the same time, the breeding values take into account the kinship structure (all “mutant” animals are assumed to be related) and other environmental and measurement factors (for more details, see Simčič et al., 2021 [17]). In statistical analyses, for each trait, the mean breeding value of 44 “mutant” animals was compared with the distribution of the mean breeding values generated by random sampling of 44 “normal” animals (10,000 times). The credibility interval was calculated for the generated distributions for each trait considered. All statistical analyses were performed using scripts written in R software [35,36].

## Data Availability

Data used for the review are available upon reasonable request to the corresponding author.
